# Molecular characterization and clinical relevance of m^6^A regulators across 33 cancer types

**DOI:** 10.1186/s12943-019-1066-3

**Published:** 2019-09-14

**Authors:** Yongsheng Li, Jun Xiao, Jing Bai, Yi Tian, Yinwei Qu, Xiang Chen, Qi Wang, Xinhui Li, Yunpeng Zhang, Juan Xu

**Affiliations:** 1 College of Bioinformatics Science and Technology, Harbin Medical University, Harbin, 150081 Heilongjiang China; 2 Key Laboratory of Tropical Translational Medicine of Ministry of Education, Hainan Medical University, Haikou, 571199 China

**Keywords:** m^6^A regulators, Pan-cancer, Genetic alterations, Cancer pathways, Survival

## Abstract

The methylation of N^6^ adenosine (m^6^A) plays a critical role in diverse biological processes. However, knowledge regarding the reconstitution of m^6^A across cancer types is still lacking. Here, we systematically analyzed the molecular alterations and clinical relevance of m^6^A regulators across > 10,000 subjects representing 33 cancer types. We found that there are widespread genetic alterations to m^6^A regulators, and that their expression levels are significantly correlated with the activity of cancer hallmark-related pathways. Moreover, m^6^A regulators were found to be potentially useful for prognostic stratification, and we identified IGF2BP3 as a potential oncogene across multiple cancer types. Our results provide a valuable resource that will guide both mechanistic and therapeutic analyses of the role of m^6^A regulators in cancer.

**Supplementary information:**

**Supplementary information** accompanies this paper at 10.1186/s12943-019-1066-3.

Methylation of N^6^ adenosine (m^6^A) is the most common type of RNA modification, and it plays crucial roles in the development and progression cancer [1, 2]. RNA methylation, similar to DNA or protein modification, is regulated by different types of regulators, including methyltransferases (‘writers’), RNA binding proteins (‘readers’), and demethylases (‘erasers’). Discovery of these different m^6^A regulators has dramatically increased our understanding of the role of RNA methylation in the regulation of gene expression [3, 4]. In addition, m^6^A perturbations mediated by these regulators have been shown to dysregulate cell death and cell proliferation, contributing to multiple different human diseases [5, 6]. A comprehensive understanding of the genetic alterations and expression perturbations underlying cancer cell heterogeneity is necessary to elucidate RNA methylation-based therapeutic targets.

In this study, we aimed to systematically characterize the molecular alterations and clinical relevance of m^6^A RNA regulators across 33 cancer types [7]. We found that there exist widespread genetic alterations (including mutations and copy number variations) in m^6^A regulators across cancer types. We also aimed to assess whether perturbations in the expression of m^6^A regulators was correlated with the activity of cancer pathways. Moreover, we explored the clinical prognostic value of m^6^A regulators, and found that m^6^A regulators are potentially useful markers for prognostic stratification. Our analysis highlights the importance of m^6^A regulators in cancer development, and lays a foundation for the development of therapeutic strategies based on RNA methylation.

## Results and discussion

### Widespread genetic alterations of m^6^A regulators across cancer types

**Fig. 1:**
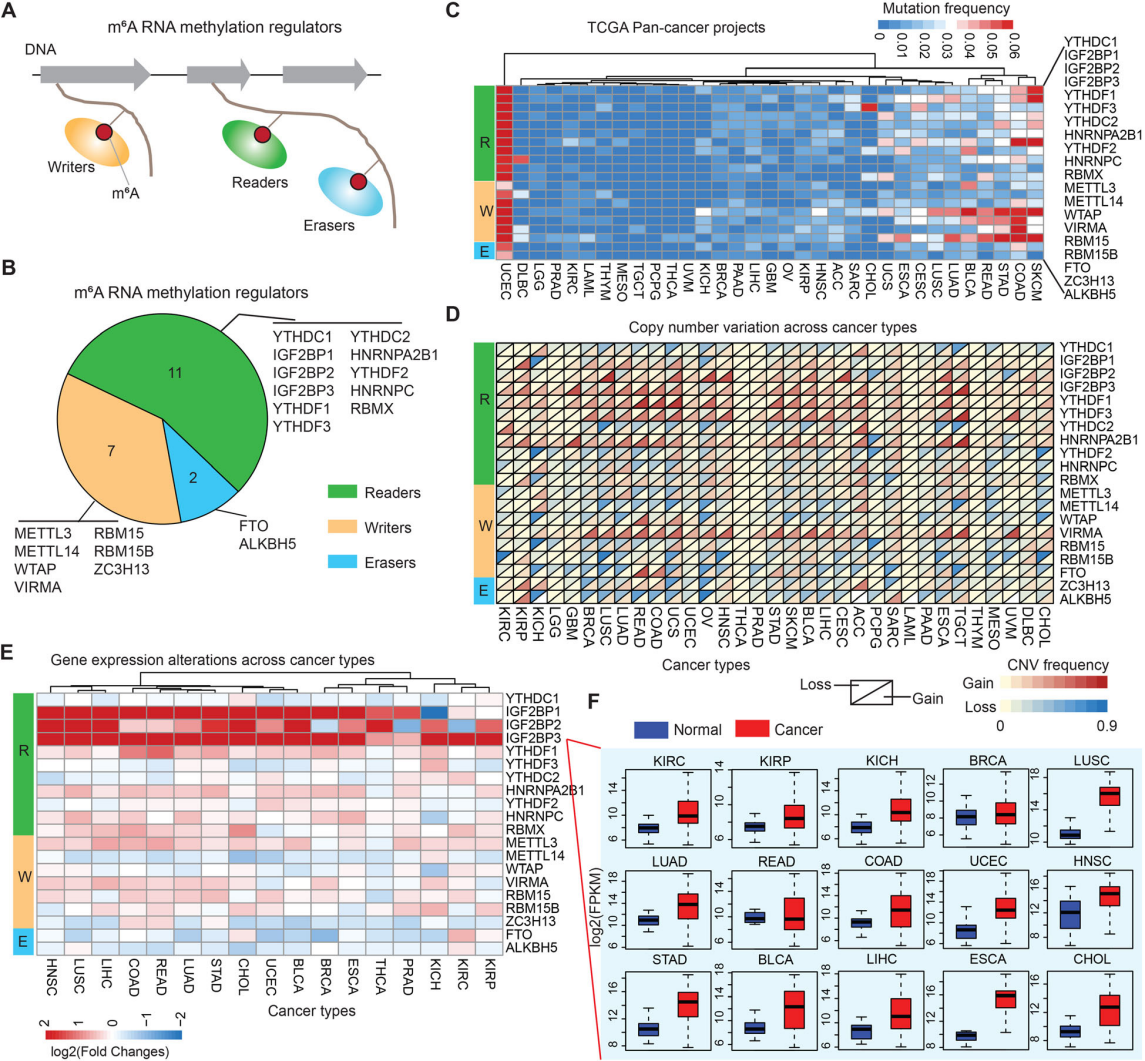
Pan-cancer genetic and expression alterations of m^6^A regulators. **a** Diagram of m6A regulators. **b** The proportion of readers, writers and erasers among m^6^A regulators. **c** The mutation frequency of m^6^A regulators across 33 cancer types. **d** The CNV alteration frequency of m^6^A regulators across cancer types. The upper part of each grid shows the deletion frequency, and the bottom part shows the amplification frequency. **e** The gene expression alterations of m^6^A regulators in 17 cancer types. The heat map shows the fold changes, with red representing up-regulated genes, and blue representing down-regulated genes. **f** Box plots showing the expression distribution of IGF2BP3 across tumor and normal samples in 15 cancer types

The numbers of m^6^A regulators have been identified, and they can be broadly classified as readers, writers, and erasers (Fig. 1a). We reviewed the literature and curated a catalog of 20 genes that function mainly as regulators of RNA methylation (Fig. 1b), including 11 readers, seven writers, and two erasers. We first determined the prevalence of m^6^A regulator alterations across 33 cancer types (Additional file 1: Table S1) by integrating data on somatic mutations and copy number variations (CNVs). The overall average mutation frequency of m^6^A regulators was low, ranging from 0.02–8.07% (Fig. 1c and Additional file 1: Table S2). Cancer types with a higher global mutation burden (such as UCEC and SKCM) also exhibited a higher mutation frequency in m^6^A regulators. We found that YTHDC1, IGF2BP1, YTHDC2 and FTO showed higher mutation frequencies (Fig. 1c). Moreover, we found that several cancer types exhibited relatively few mutations in m^6^A regulators compared to other cancers, such as PCPG, THCA and UVM. We next collected the mutation data for 967 cell lines across 23 cancers from the Cancer Cell Line Encyclopedia (CCLE) and 652 cell lines across 22 cancers from the Genomics of Drug Sensitivity in Cancer (GDSC) database. We found that the writers had relatively high mutation frequencies across cancer types (Additional file 2: Figure S1).

We next investigated the CNV alteration frequency for all m^6^A regulators, and found that CNV alterations are prevalent. IGF2BP1/2/3, YTHDF1/3, HNRNPA2B1, and VIRMA showed widespread CNV amplification across cancer types (Fig. 1d and Additional file 1: Table S3). In contrast, YTHDC1/2, METTL14, RBM15B, and ALKBH5 had prevalent CNV deletions (Fig. 1d and Additional file 1: Table S4). There were also prevalent CNV alterations in m^6^A regulators across cell lines (Additional file 2: Figure S2). An intriguing question is whether these genetic alterations affect the expression of m^6^A regulators. We therefore explored the expression perturbations of m^6^A regulators across 17 cancer types with at least five normal controls. We found that CNV alterations are most likely one of the prominent mechanisms leading to perturbations in the expression of m^6^A regulators (Fig. 1e). The m^6^A regulators with CNV amplification showed significantly higher expression in cancer cells when compared to normal cells (e.g. IGF2BP1 and IGF2BP3), while the regulators with CNV deletion showed significantly lower expression (e.g. METTL14 and ALKBH5). In particular, we found that IGF2BP3 showed significantly higher expression in 15 cancer types (Fig. 1f). Moreover, we analyzed the expression of m6A regulators across another ~ 7400 samples, representing 11 cancer types, and found that IGF2BP3 also showed higher expression in cancer cells (Additional file 2: Figure S3). These results reveal a highly heterogeneous genetic and expression alteration landscape of m^6^A regulators across cancer types, suggesting that m^6^A regulator dysregulation is of importance in different cancer contexts.

### Oncogenic pathways regulated by m^6^A regulators

**Fig. 2:**
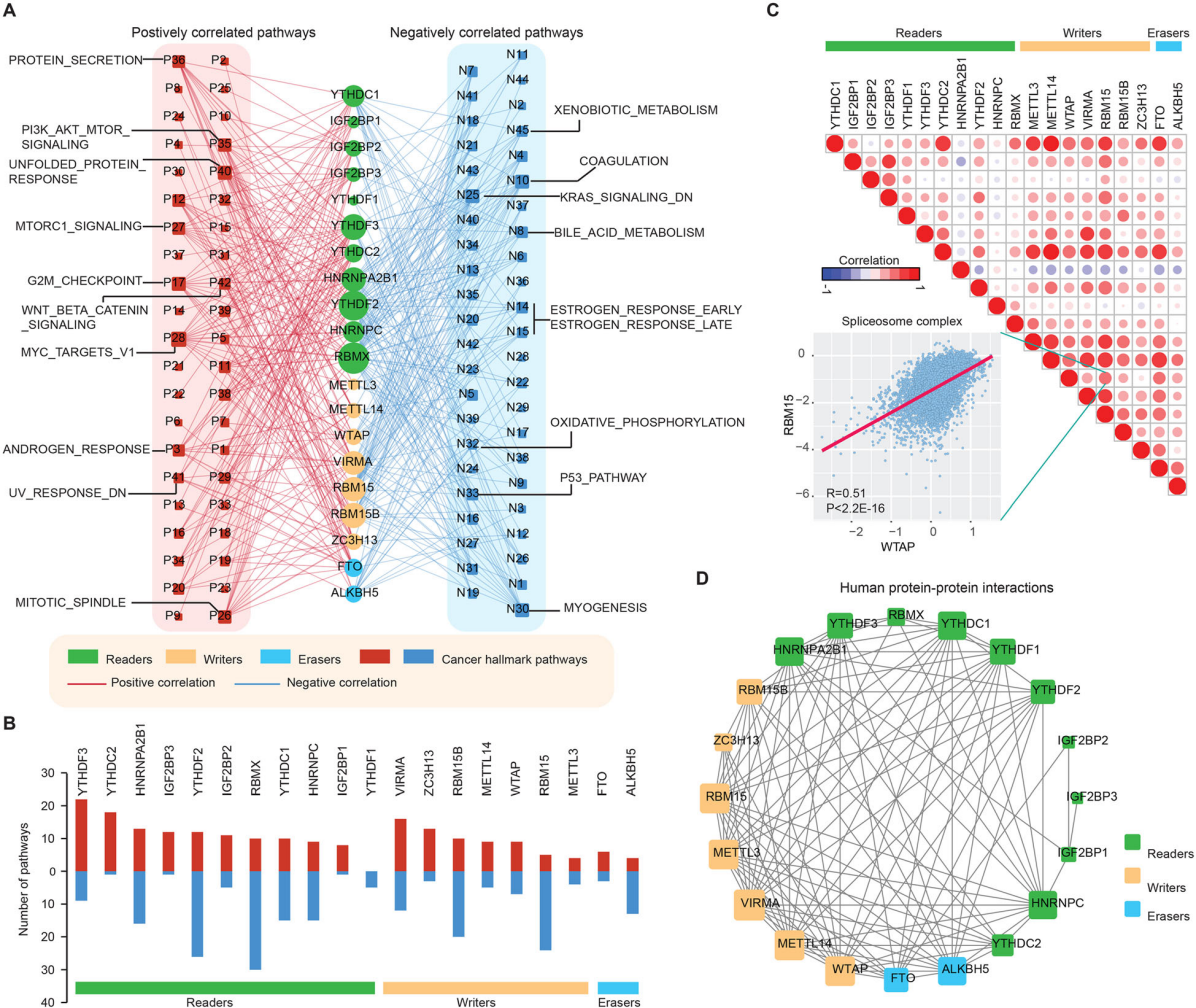
m^6^A regulators are correlated with the activation and inhibition of cancer pathways. **a** Network diagram demonstrating the correlation between m^6^A regulators and cancer pathways. Red represents a positive correlation, and blue represents a negative correlation. The size of the nodes corresponds to the number of links. **b** The number of pathways is correlated with individual m^6^A regulators. The upper panel is for positively correlated pathways, and the bottom panel is for negatively correlated pathways. **c** Correlation among the expression of m^6^A regulators. The scatter plot shows the correlation between WTAP and RBM15. **d** The protein-protein interactions among m^6^A regulators

To further understand the molecular mechanisms by which m^6^A regulators are involved in cancer, we examined the correlation between the expression of individual m^6^A regulators and the activity of 50 cancer hallmark-related pathways (Additional file 2: Methods). We found that the expression of m^6^A regulators is correlated with the activation or inhibition of multiple oncogenic pathways (Fig. 2a and Additional file 1: Table S5). The expression of YTHDF2, RBMX, and RBM15 was correlated with a higher number of activated pathways, such as the PI3K-AKT-MTOR, G2M checkpoint, and P53 pathways. In particular, we found that the m^6^A reader YTHDF3 was correlated with the activation of several pathways (Fig. 2b), including protein secretion (in 18/33 cancers), androgen response (in 8/33 cancers) and the TGFb signaling pathway (in 6/33 cancers). In contrast, we found that the expression of HNRPA2B1 was correlated with the activity of several cancer pathways (Fig. 2b), including the E2F, G2M checkpoint, and KRAS signaling pathways. These correlations were further validated using HNRPA2B1 knockdown experiments (Additional file 2: Figure S4). In addition, different readers, writers, or erasers were associated with distinct cancer pathway alterations, suggesting different functional effects of m^6^A regulators within the same functional class.

Moreover, genes do not function in isolation, and evidence has shown that collaboration among writers, erasers, and readers exists in the context of cancer [8, 9]. We thus investigated the co-occurrence of genetic alterations and expression correlation among m^6^A regulators. We found not only that genes within the same functional class showed significant co-occurrences of genetic alterations and highly correlated expression patterns (Additional file 2: Figure S5), but that a high correlation also existed among writers, erasers, and readers (Fig. 2c). For instance, the reader YTHDC1 was significantly correlated with writers, such as METTL3 and METTL14. We also found that there were higher correlations among genes in the same protein complex, such as RBM15 and WTAP of the spliceosome complex (Fig. 2c, R = 0.51 and *p*-value < 2.2E-16). Moreover, we found that these writers, erasers, and readers interacted with each other frequently in protein-protein interaction networks (Fig. 2d). There was an especially high number of interactions among the writers. Taken together, these results suggest that cross-talk among the writers, readers, and erasers of RNA methylation, play critical roles in the development and progression of different types of cancers.

### Clinical relevance of m^6^A regulators across cancer types

**Fig. 3:**
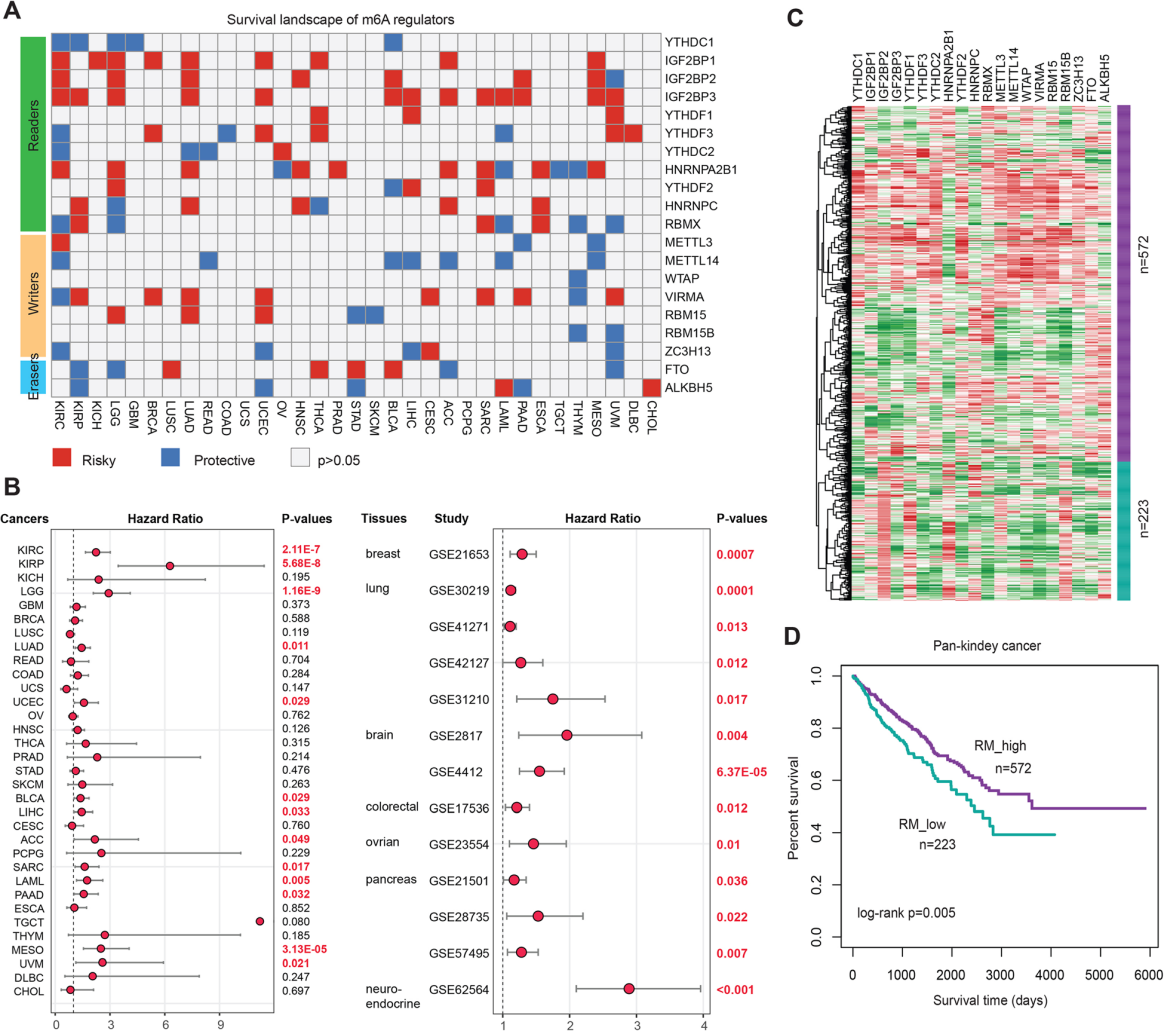
Clinical relevance of m^6^A regulators across cancer types. **a** Summary of the correlation between expression of m^6^A regulators and patient survival. Red represents a higher expression of m^6^A regulator associated with worse survival, and blue represents an association with better survival. Only *p* values < 0.05 are shown. **b** The distribution of hazard ratios across different cancer types. **c** Heat map showing the clustering for kidney cancer patients based on the expression of m^6^A regulators. **d** Kaplan-Meier survival plot of patients grouped by global expression pattern of m^6^A regulators

The prevalent genetic and expression alterations in m^6^A regulators in various types of cancers, may provide important insight into translational medicine developments. First, we found that m6A regulators were more likely to be essential genes across cell lines, suggesting that they play critical roles in cell growth (Additional file 2: Figure S6). Next, we focused on m^6^A regulators that showed significant association with patient survival across the 33 cancer types. We found that all of the m^6^A regulators were associated with the overall survival of patients in at least one cancer type (Fig. 3a). Several m^6^A regulator genes showed oncogenic features, such as IGF2BP1 and IGF2BP3, and higher expression of these genes was associated with worse survival across cancer types. In particular, high expression of IGF2BP3 was correlated with worse survival in 13 cancer types (Fig. 3b), including KIRC (log-rank *p* = 2.11E-7), KIRP (log-rank *p* = 5.68E-8), and LGG (log-rank *p* = 1.16E-9). Moreover, we collected another 13 datasets across seven tissues from Gene Expression Omnibus (GEO), and found that high expression of IGF2BP3 was associated with poor patient survival (Fig. 3b and Additional file 2: Figure S7). These observations indicate that IGF2BP3 might function as an oncogene across cancer types. In contrast, we found that several m^6^A regulators also showed features of tumor suppressors, such as METTL14. Higher expression of METTL14 was significantly associated with better survival in seven cancer types (Fig. 3a and Additional file 2: Figure S8).

Moreover, we found more m^6^A regulators that were correlated with patient survival in two types of kidney cancer. We thus explored whether the expression of m^6^A regulators could contribute to the stratification of kidney cancer. Based on the global expression pattern of m^6^A regulators, we identified two subgroups of kidney cancer patients (Fig. 3c). The first subgroup consisted of 572 patients that showed higher expression of m^6^A regulators (RM-high), and the second of 223 patients with low expression (RM-low). Compared to the RM-low subgroup, patients in the RM-high subgroup had significantly better survival rates (Fig. 3d, log-rank *p* = 0.005). To further understand the clinical implications of m^6^A regulators, we examined the correlation between m^6^A regulators and 150 clinically actionable genes [10], and observed that m^6^A regulators frequently interacted with these genes (Additional file 2: Figure S9). Moreover, we manually searched the literature and found that the majority of these regulators have been found to play critical roles in cell growth, proliferation, and metastasis (Additional file 1: Table S6). However, the function of several regulators still require further validation in low throughput experiments. Together, these results suggest a diverse potential of m^6^A regulators in the prognostic stratification of specific types of cancer and in the development of novel treatment strategies.

## Conclusions

We have demonstrated the prevalent genetic and expression alterations of RNA methylation regulators across cancer types. These m^6^A regulators are tightly correlated with the activation and inhibition of cancer pathways, and are also correlated with prognostically relevant tumor subtypes. In conclusion, this systematic analysis of the landscape of molecular alterations and clinical relevance of m^6^A regulators lays a critical foundation for understanding the dysregulation of RNA methylation. It will also provide insights into the development of related therapeutic targets.

## Supplementary information

**Additional file 1: Table S1.** The 33 cancer types in TCGA pancancer project. **Table S2.** The mutation frequency of m6A regulators across 33 cancer types. **Table S3.** The CNV amplification frequency of m6A regulators across 33 cancer types. **Table S4**. The CNV deletion frequency of m6A regulators across 33 cancer types. **Table S5.** The correlation of m6A regulations and cancer pathways. **Table S6.** Literature curation of the function for m6A regulators. (XLSX 62 kb)

**Additional file 2:** Supplementary materials and methods, and supplementary Figure S1-S9. **Figure S1.** Mutation frequency distribution of m6A regulators across different cancer types. **Figure S2.** CNV alterations of m6A regulators across cell lines in different cancer types. **Figure S3.** Gene expression of m6A regulators across cancer types. **Figure S4. **Pathways potentially regulated by HNRPA2B1. **Figure S5.** Co-occurrence of genetic alterations of regulators across cancer types. **Figure S6.** Function of m6A regulators in cell growth. **Figure S7.** Kaplan-Meier survival plots of patients grouped by the expression of IGF2BP3 in individual cancer types. **Figure S8.** Kaplan-Meier survival plots of patients grouped by the expression of METTL14 in individual cancer types. **Figure S9.** Protein-protein interactions among m6A regulators and clinical actionable genes obtained from STRING database. (DOCX 2661 kb)

## Data Availability

The gene expression profiles and clinical data can be found at the GDC portal (https://portal.gdc.cancer.gov/). The mutation data can be downloaded from Synapse (https://www.synapse.org/#!Synapse:syn4977808). The copy number data were downloaded from Broad GDAC Firehose (https://gdac.broadinstitute.org/). Software and resources used for the analyses are described in each method section. All results generated in this study can be found in supplementary tables.

## Abbreviations

CNV: Copy number variation
KIRC: Kidney Renal Clear Cell Carcinoma
KIRP: Kidney renal papillary cell carcinoma
LGG: Brain low grade glioma
m^6^A: Methylation of N^6^ adenosine
PCPG: Pheochromocytoma and Paraganglioma
SKCM: Skin Cutaneous Melanoma
THCA: Thyroid Cancer
UCEC: Uterine Corpus Endometrial Carcinoma
UVM: Uveal Melanoma
