# Supplementary material for: Molecular characterization and clinical relevance of m6A regulators across 33 cancer types
Source: Mol Cancer. 2019 Sep 14;18:137. doi: 10.1186/s12943-019-1066-3 (PMC6744659; doi:10.1186/s12943-019-1066-3)
Supplement: Supplementary file 2 — Additional file 2: Supplementary materials and methods, and supplementary Figure S1-S9. Figure S1. Mutation frequency distribution of m6A regulators across different cancer types. Figure S2. CNV alterations of m6A regulators across cell lines in different cancer types. Figure S3. Gene expression of m6A regulators across cancer types. Figure S4. Pathways potentially regulated by HNRPA2B1. Figure S5. Co-occurrence of genetic alterations of regulators across cancer types. Figure S6. Function of m6A regulators in cell growth. Figure S7. Kaplan-Meier survival plots of patients grouped by the expression of IGF2BP3 in individual cancer types. Figure S8. Kaplan-Meier survival plots of patients grouped by the expression of METTL14 in individual cancer types. Figure S9. Protein-protein interactions among m6A regulators and clinical actionable genes obtained from STRING database. (DOCX 2661 kb) [file 12943_2019_1066_MOESM2_ESM.docx]

**Supplementary materials and methods**

**Collection of m^6^A regulators**

We collected 20 m^6^A regulators from recently published review papers [1, 2], including 11 readers, seven writers, and two erasers. All these gene symbols were converted into Ensemble gene IDs and HGNC symbols by manually curated from GeneCards (https://www.genecards.org/).

**Genome-wide omics data across 33 cancer types**

The results in our analysis are based upon omics datasets generated by TCGA Research Network (<http://cancergenome.nih.gov/>). We totally analyzed 33 different TCGA projects, each project represents a specific cancer type, including KIRC, kidney renal clear cell carcinoma; KIRP, kidney renal papillary cell carcinoma; KICH, kidney chromophobe; LGG, brain lower grade glioma; GBM, glioblastoma multiforme; BRCA, breast cancer; LUSC, lung squamous cell carcinoma; LUAD, lung adenocarcinoma; READ, rectum adenocarcinoma; COAD, colon adenocarcinoma; UCS, uterine carcinosarcoma; UCEC, uterine corpus endometrial carcinoma; OV, ovarian serous cystadenocarcinoma; HNSC, head and neck squamous carcinoma; THCA, thyroid carcinoma; PRAD, prostate adenocarcinoma; STAD, stomach adenocarcinoma; SKCM, skin cutaneous melanoma; BLCA, bladder urothelial carcinoma; LIHC, liver hepatocellular carcinoma; CESC, cervical squamous cell carcinoma and endocervical adenocarcinoma; ACC, adrenocortical carcinoma; PCPG, pheochromocytoma and paraganglioma; SARC, sarcoma; LAML, acute myeloid leukemia; PAAD, pancreatic adenocarcinoma; ESCA, esophageal carcinoma; TGCT, testicular germ cell tumors; THYM, thymoma; MESO, mesothelioma; UVM, uveal melanoma; DLBC, lymphoid neoplasm diffuse large b-cell lymphoma; CHOL, cholangiocarcinoma.

All the somatic mutations were obtained from the publicly available TCGA MAF file (“MC3”) which covers >10,000 patients [3]. This dataset was directly downloaded from Synapse under the number of syn7214402. Six calling methods and numbers of filters were applied. The copy number variation data were downloaded from Broad GDAC Firehose (https://gdac.broadinstitute.org/). GISTIC was used to identify genomic regions that are significantly gained or lost across a set of tumors [4]. RNA-seq data were obtained from the TCGA project via the R-package “TCGAbiolinks” [5], which is specifically developed for integrative analysis with GDC data. We downloaded the Fragments Per Kilobase of transcript per Million mapped reads (FPKM)-based gene expression for 33 types of cancer. The clinical information for patients of 33 cancer types were downloaded from TCGA project via the R-package “TCGAbiolinks”, including the survival status, stages, grades, survival time.

**Genomic, transcriptomic data of m6A regulators across cell lines and cancers**

Genome-wide mutation data across cell lines were collected from the Broad Institute Cancer Cell Line Encyclopedia (CCLE) and the Genomics of Drug Sensitivity in Cancer database [6, 7]. These cell lines were classified into different cancer types based on their annotations. In total, there were 967 cell lines across 23 cancer types from CCLE and 652 cell lines across 22 cancer types from GDSC. The mutation frequency of m6A regulators in each cancer type was defined as the proportion of cell lines with the regulator mutations. In addition, we also downloaded the copy number variation data for cell lines from CCLE and GDSC. There were 971 cell lines across 24 cancer types in CCLE and 649 cell lines in GDSC with CNV data. We calculated the CNV frequency in each cancer types as the proportion of cell lines with CNV amplification and deletion.

To validate the expression of m6A regulators across cancer types, we collected gene expression data across ~7400 samples representing 11 cancer types. These data were collected from Gene Expression Omnibus (GEO). To minimize inter-platform variation, only datasets generated from the Affymetrix Human Genome U133 Plus 2.0 Array were processed to develop the meta-dataset [8]. Each dataset was preprocessed with RMA normalization, merged, and batch effect-corrected via Combat method [9].

**Identification of differentially expressed genes**

To identify differentially expressed genes in each cancer type, we used the Wilcox’s rank sum test to identify differentially expressed genes. The p-values were adjusted by BH method. Genes with adjusted p-values <0.01 and at least two-fold changes in expression were identified as differentially expressed genes in each cancer type.

**Oncogenic pathway activity across cancer types**

To calculate the activity of cancer hallmark-related pathways, we first transformed the FPKM-based gene expression to Z-score. This is performed by zFPKM package [10]. The normalized gene expression were subjected to Gene Set Variation Analysis (GSVA), which is a non-parametric, unsupervised method for estimating variation of gene set enrichment through the samples of an expression data set [11]. To identify the m^6^A regulators that were correlated with activation or inhibition of pathway, we calculated the Pearson Correlation Coefficient (PCC) between expression of m^6^A regulators and pathway activity. The regulator-pathway pairs with |PCC|>0.5 and adjusted p-value<0.01 were identified as significantly correlated m^6^A regulators.

**Validation of m6A-pathway correlation**

To validate the m6A-pathway correlation, we manually curated the GEO database and collected m6A perturbed gene expression data. Here, we focused one data for transcriptional profiling of MDA-MB-231 samples taken at 0, 1, 2 and 4hr time points, comparing the siRNA-mediated HNRPA2B1 knock-downs versus mock-transfected controls [12]. For each gene, we calculated the perturbed score which was defined as

$$S=-log10\left( p \right)*sign(Fold-change)$$

Where p-value is the paired t test between knock-down and controls, and fold-changes is the ratio between average expressions in knock-downs vs controls. All the genes were ranked by this score and subjected to pre-ranked Gene Set Enrichment Analysis (GSEA). The cancer hallmark-related functions were considered [13].

**Cross talks among m^6^A regulators**

We calculated the PCC among m^6^A regulators based on gene expression across all cancer types. The PCCs among m^6^A regulators were visualized by corrplot package in R program (<https://github.com/taiyun/corrplot>). In addition, we identified the protein-protein interactions among m^6^A regulators based on STRING interaction database [14]. The 150 clinical actionable genes were obtained from one of recent publication [15]. Only the interactions between m6A regulators and actionable genes were visualized by Cytoscape [16].

**Clinical relevance of m^6^A regulators**

To explore whether the expression of m^6^A regulators was associated with patient survival, we divided all the patients into two groups based on the median expression of each m^6^A regulator. The log-rank test was used to test the difference survival rates between two groups. This process was performed by the survival package in R program (https://cran.r-project.org/web/packages/survival/index.html). The p-values <0.05 were considered as significant.

**Validating the clinical association of IGF2BP3**

We validated the clinical association of IGF2BP3 based on datasets from PROGgeneV2 [17] and LCE [18]. Patients were also divided into two groups based on the median expression of IGF2BP3, and the survival difference were tested by log-rank test. The hazard ratio and 95% confidence level were calculated and plotted by ggplot2 package.

**The functions of m6A regulators in cell growth**

Genome-scale CRISPR-Cas9 screens in 324 human cancer cell lines from 30 cancer types were collected from literature [19]. We calculated the proportion of cell lines that each regulator was observed as essential gene. In addition, we randomly selected the same number of genes as the regulators and re-calculated the proportion of cell lines. This process was repeated 1000 times and the p-value is defined as the percent of random conditions that with a higher average proportion than observed.

**Supplementary references**

1. Yang Y, Hsu PJ, Chen YS, Yang YG: **Dynamic transcriptomic m(6)A decoration: writers, erasers, readers and functions in RNA metabolism.** *Cell Res* 2018, **28:**616-624.

2. Pinello N, Sun S, Wong JJ: **Aberrant expression of enzymes regulating m(6)A mRNA methylation: implication in cancer.** *Cancer Biol Med* 2018, **15:**323-334.

3. Zhang Y, Kwok-Shing Ng P, Kucherlapati M, Chen F, Liu Y, Tsang YH, de Velasco G, Jeong KJ, Akbani R, Hadjipanayis A, et al: **A Pan-Cancer Proteogenomic Atlas of PI3K/AKT/mTOR Pathway Alterations.** *Cancer Cell* 2017, **31:**820-832 e823.

4. Mermel CH, Schumacher SE, Hill B, Meyerson ML, Beroukhim R, Getz G: **GISTIC2.0 facilitates sensitive and confident localization of the targets of focal somatic copy-number alteration in human cancers.** *Genome Biol* 2011, **12:**R41.

5. Colaprico A, Silva TC, Olsen C, Garofano L, Cava C, Garolini D, Sabedot TS, Malta TM, Pagnotta SM, Castiglioni I, et al: **TCGAbiolinks: an R/Bioconductor package for integrative analysis of TCGA data.** *Nucleic Acids Res* 2016, **44:**e71.

6. Ghandi M, Huang FW, Jane-Valbuena J, Kryukov GV, Lo CC, McDonald ER, 3rd, Barretina J, Gelfand ET, Bielski CM, Li H, et al: **Next-generation characterization of the Cancer Cell Line Encyclopedia.** *Nature* 2019, **569:**503-508.

7. Yang W, Soares J, Greninger P, Edelman EJ, Lightfoot H, Forbes S, Bindal N, Beare D, Smith JA, Thompson IR, et al: **Genomics of Drug Sensitivity in Cancer (GDSC): a resource for therapeutic biomarker discovery in cancer cells.** *Nucleic Acids Res* 2013, **41:**D955-961.

8. Bin Lim S, Chua MLK, Yeong JPS, Tan SJ, Lim WT, Lim CT: **Pan-cancer analysis connects tumor matrisome to immune response.** *NPJ Precis Oncol* 2019, **3:**15.

9. Leek JT, Johnson WE, Parker HS, Jaffe AE, Storey JD: **The sva package for removing batch effects and other unwanted variation in high-throughput experiments.** *Bioinformatics* 2012, **28:**882-883.

10. Hart T, Komori HK, LaMere S, Podshivalova K, Salomon DR: **Finding the active genes in deep RNA-seq gene expression studies.** *BMC Genomics* 2013, **14:**778.

11. Hanzelmann S, Castelo R, Guinney J: **GSVA: gene set variation analysis for microarray and RNA-seq data.** *BMC Bioinformatics* 2013, **14:**7.

12. Goodarzi H, Najafabadi HS, Oikonomou P, Greco TM, Fish L, Salavati R, Cristea IM, Tavazoie S: **Systematic discovery of structural elements governing stability of mammalian messenger RNAs.** *Nature* 2012, **485:**264-268.

13. Subramanian A, Tamayo P, Mootha VK, Mukherjee S, Ebert BL, Gillette MA, Paulovich A, Pomeroy SL, Golub TR, Lander ES, Mesirov JP: **Gene set enrichment analysis: a knowledge-based approach for interpreting genome-wide expression profiles.** *Proc Natl Acad Sci U S A* 2005, **102:**15545-15550.

14. Szklarczyk D, Franceschini A, Wyder S, Forslund K, Heller D, Huerta-Cepas J, Simonovic M, Roth A, Santos A, Tsafou KP, et al: **STRING v10: protein-protein interaction networks, integrated over the tree of life.** *Nucleic Acids Res* 2015, **43:**D447-452.

15. Li J, Han L, Roebuck P, Diao L, Liu L, Yuan Y, Weinstein JN, Liang H: **TANRIC: An Interactive Open Platform to Explore the Function of lncRNAs in Cancer.** *Cancer Res* 2015, **75:**3728-3737.

16. Shannon P, Markiel A, Ozier O, Baliga NS, Wang JT, Ramage D, Amin N, Schwikowski B, Ideker T: **Cytoscape: a software environment for integrated models of biomolecular interaction networks.** *Genome Res* 2003, **13:**2498-2504.

17. Goswami CP, Nakshatri H: **PROGgeneV2: enhancements on the existing database.** *BMC Cancer* 2014, **14:**970.

18. Cai L, Lin S, Girard L, Zhou Y, Yang L, Ci B, Zhou Q, Luo D, Yao B, Tang H, et al: **LCE: an open web portal to explore gene expression and clinical associations in lung cancer.** *Oncogene* 2019, **38:**2551-2564.

19. Behan FM, Iorio F, Picco G, Goncalves E, Beaver CM, Migliardi G, Santos R, Rao Y, Sassi F, Pinnelli M, et al: **Prioritization of cancer therapeutic targets using CRISPR-Cas9 screens.** *Nature* 2019, **568:**511-516.


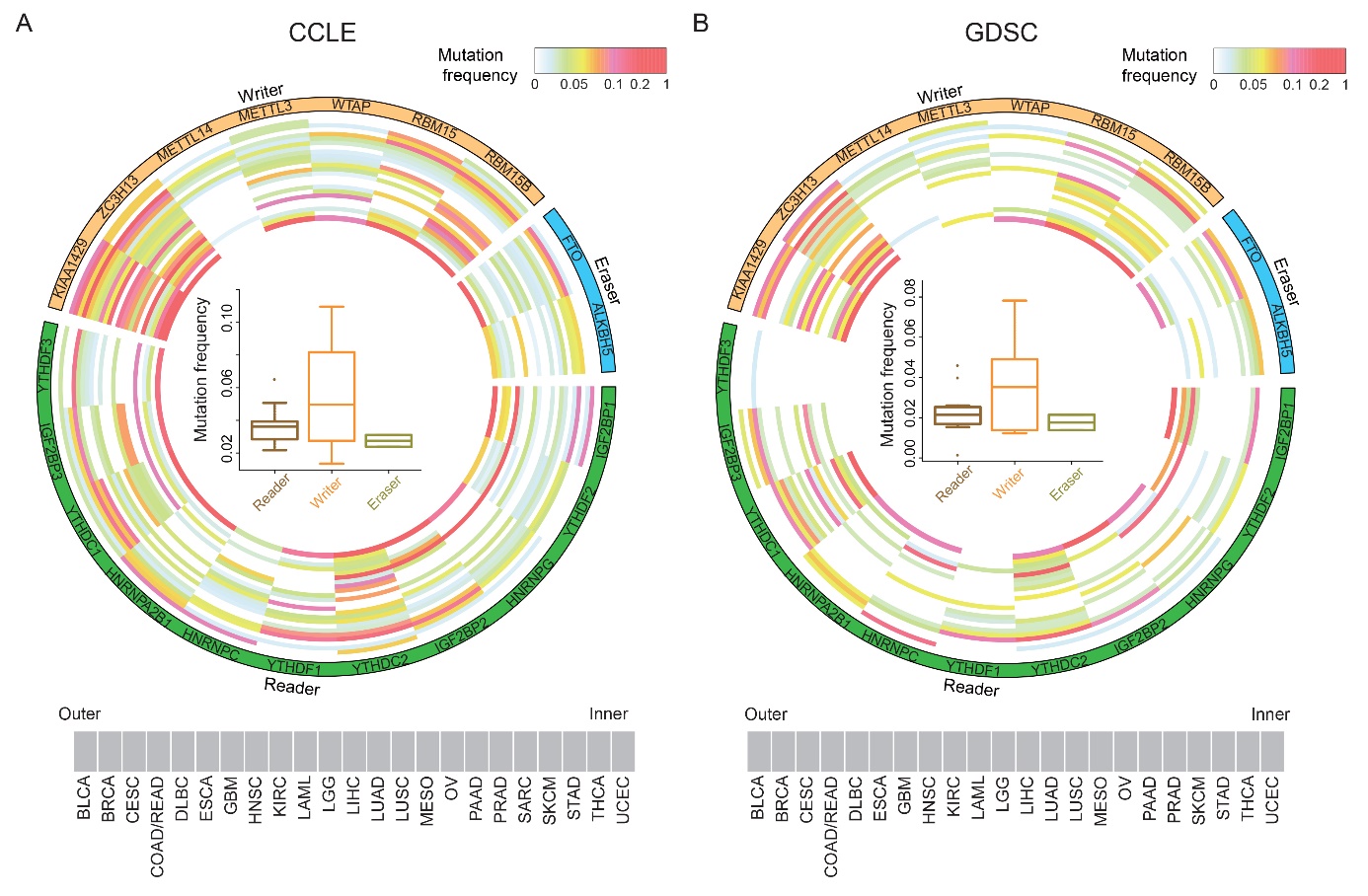


**Supplementary Fig. 1. Mutation frequency distribution of m6A regulators across different cancer types.** Left circos plot (A) showing the mutation frequency of m^6^A regulators in CCLE, and right circos (B) showing the mutation frequency in GDSC. Each circos represents one cancer types, which were shown in the bottom panel. The inner boxplots showing the pan-cancer frequencies for readers, writers and erasers.


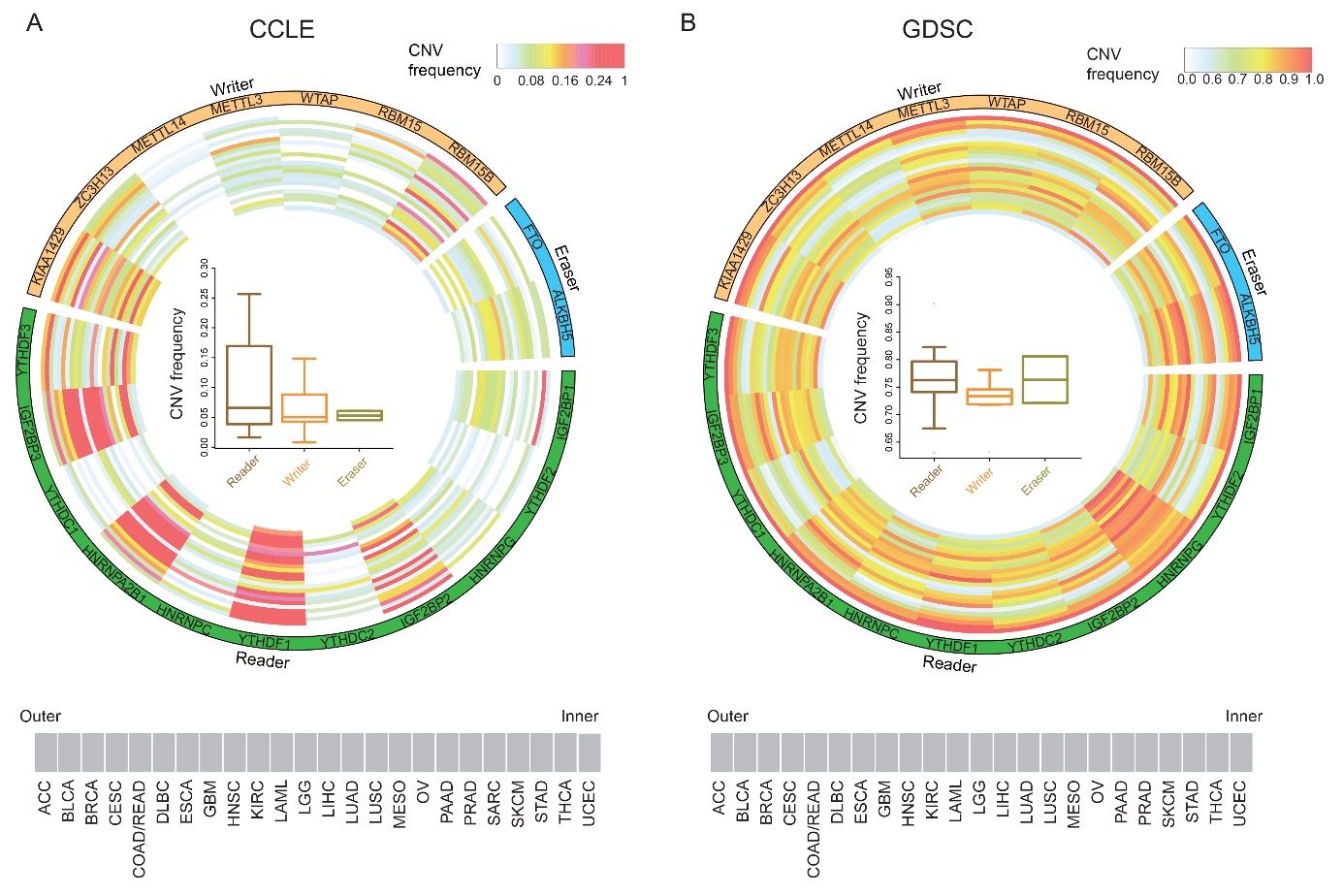


**Supplementary Fig. 2. CNV alterations of m6A regulators across cell lines in different cancer types.** Left circos plot (A) showing the CNV frequency of m^6^A regulators in CCLE, and right circos (B) showing the CNV frequency in GDSC. Each circos represents one cancer types, which were shown in the bottom panel. The inner boxplots showing the pan-cancer CNV frequencies for readers, writers and erasers.


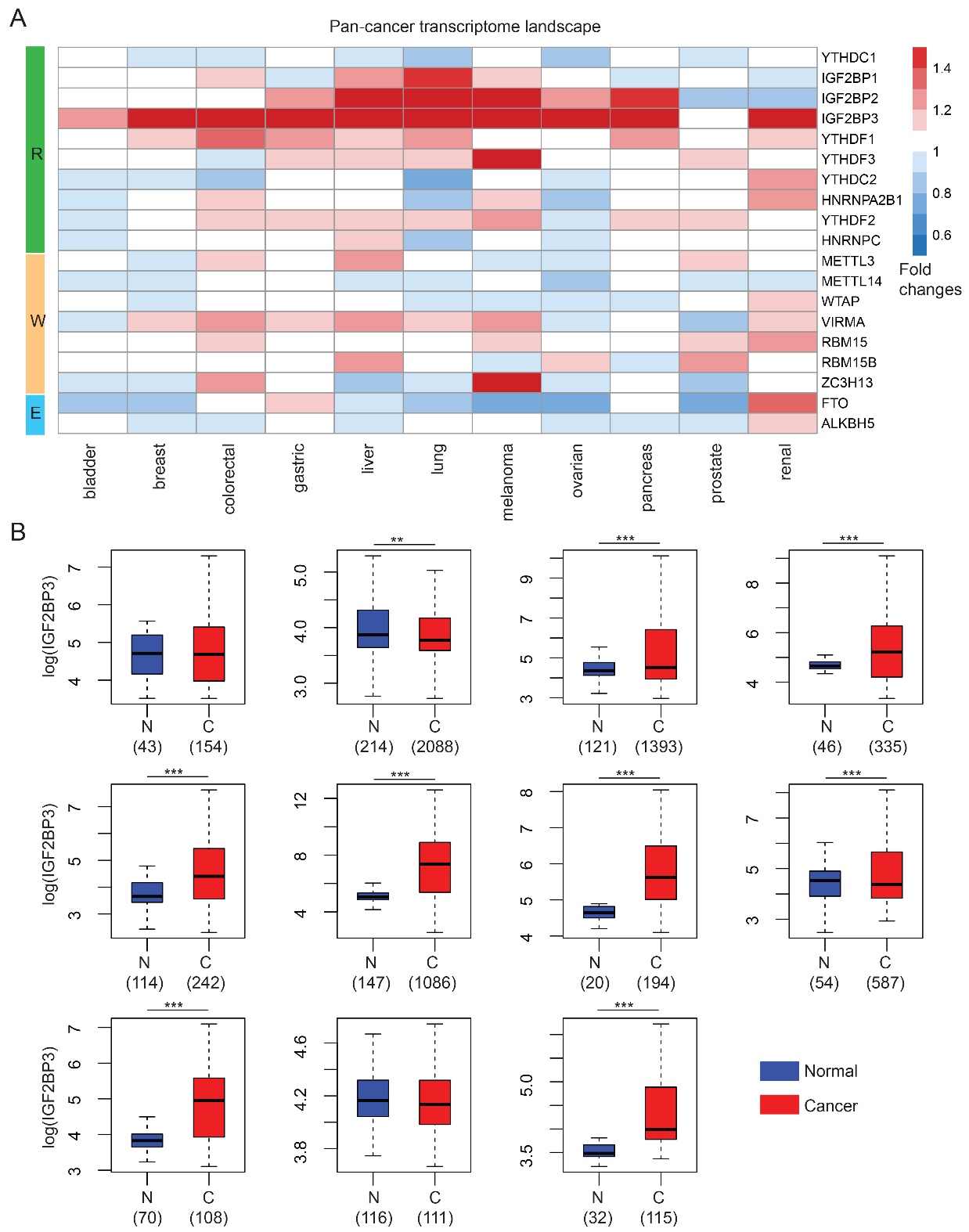


**Supplementary Fig. 3. Gene expression of m6A regulators across cancer types.**

**A,** Gene expression alterations of m^6^A regulators in 11 cancer types. Heat map showing the fold changes, with red representing up-regulated genes, and blue representing down-regulated genes. **B,** Box plots showing expression distribution of IGF2BP3 across tumor and normal samples in 11 cancer types.


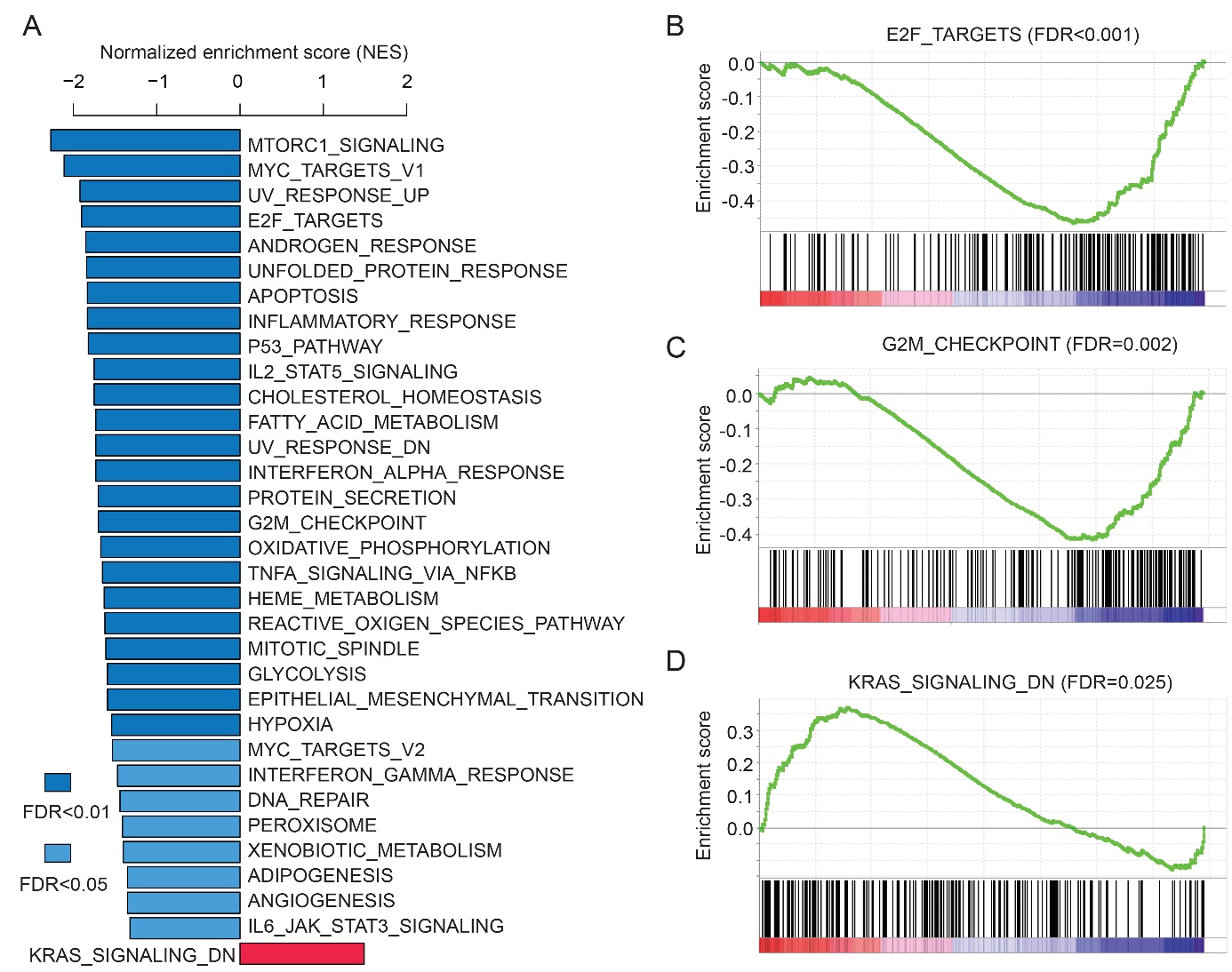


**Supplementary Fig. 4. Pathways potentially regulated by HNRPA2B1.**

**A,** Distribution of normalized enrichment scores for pathways. The pathways colored in blue was the depleted pathways in HNRPA2B1 knockout, while the red one is enriched pathway. **B-D,** GSEA-enrichment plot of the representative gene sets. (B), E2F targets; (C), G2M checkpoint; (D), KRAS signaling pathway.


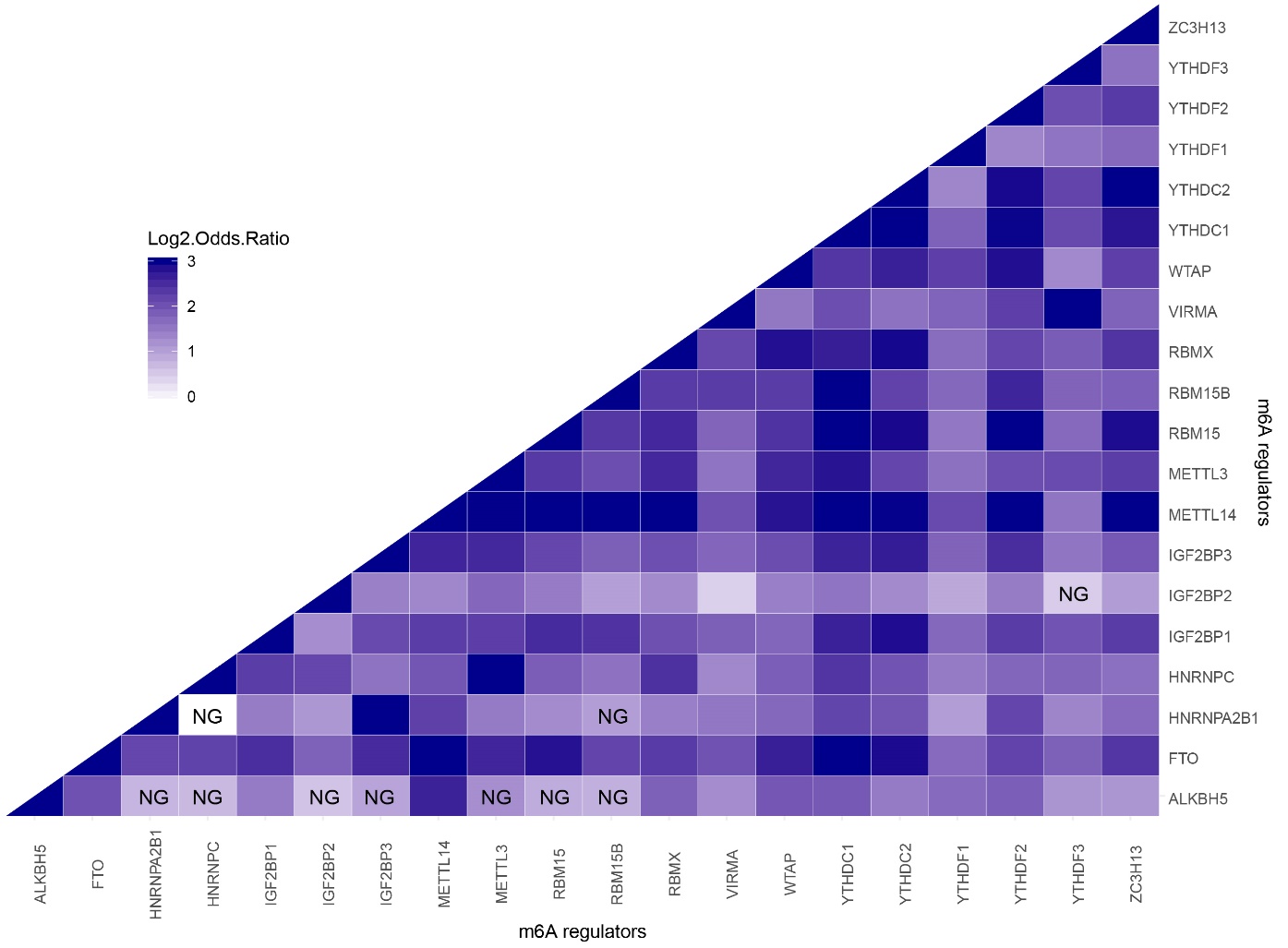


**Supplementary Fig. 5. Co-occurrence of genetic alterations of regulators across cancer types.** The log2(odds ratio) were colored as a heat map, and p-values >0.05 were indicated as NG.


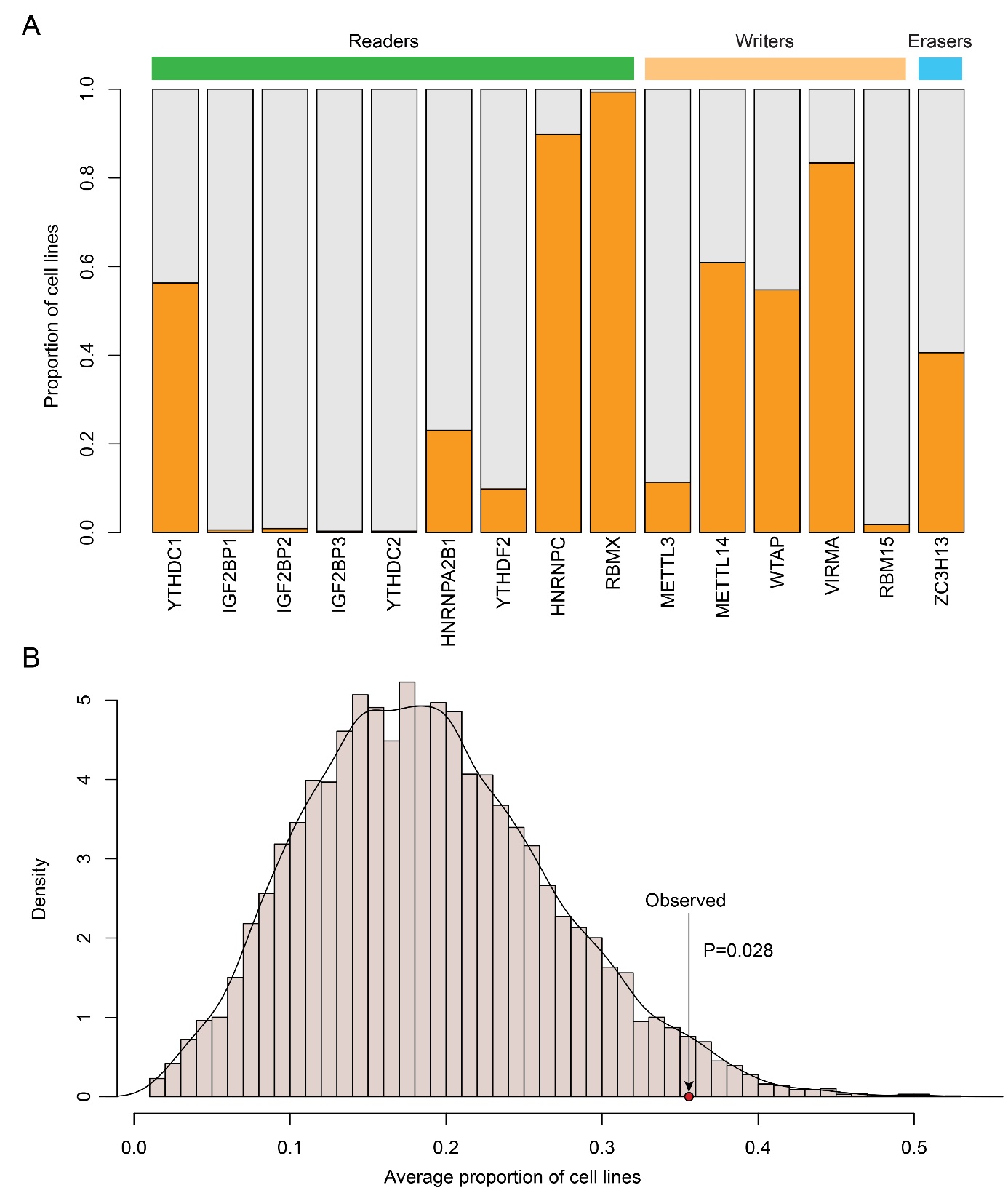


**Supplementary Fig. 6. Function of m6A regulators in cell growth.** A, The proportion of cell lines that each regulator was identified as essential gene. B, Bar plot showing the distribution of average proportion of cell lines for all regulators. Red dot indicates the observed proportion.


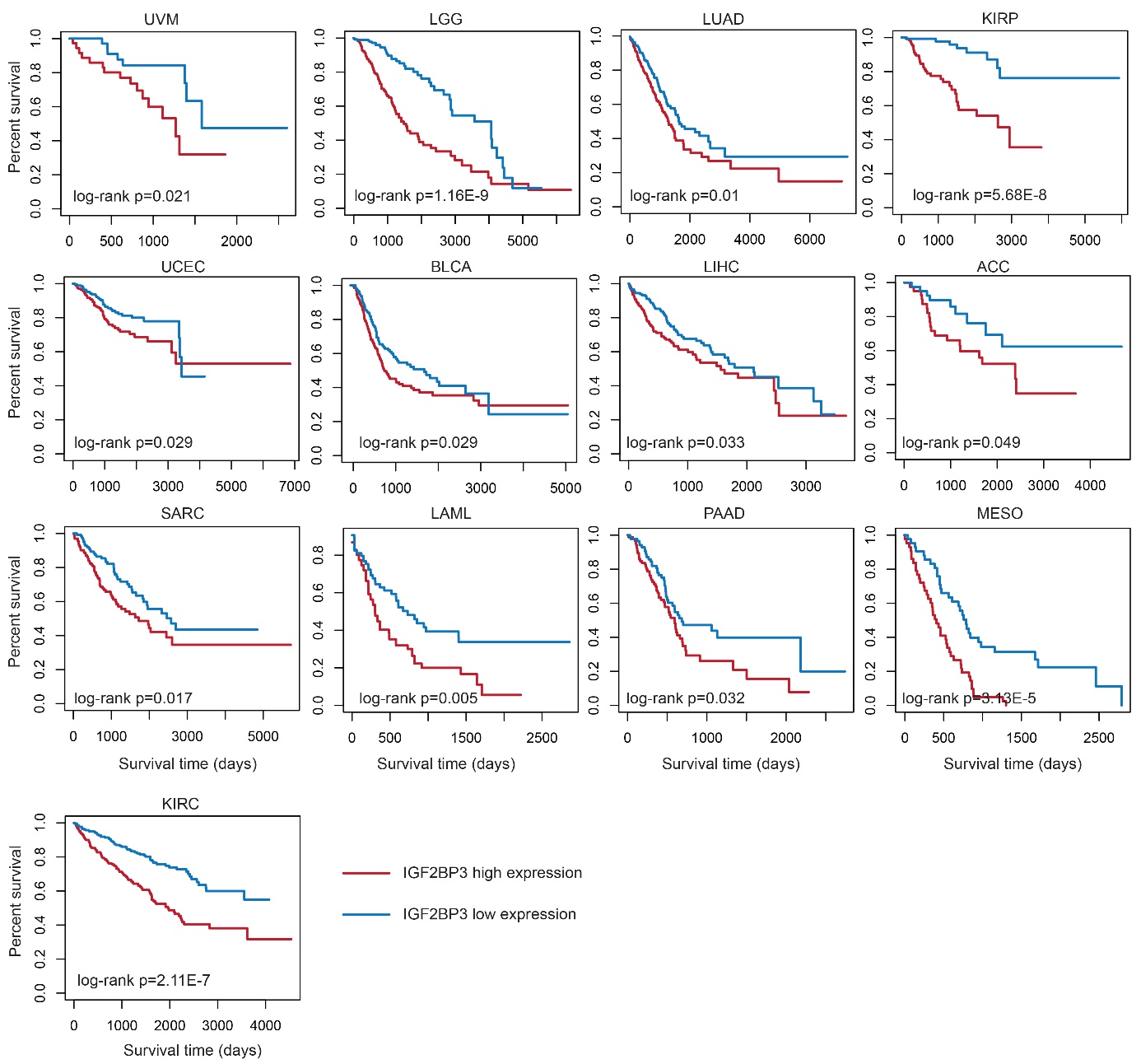


**Supplementary Fig. 7. Kaplan-Meier survival plots of patients grouped by the expression of IGF2BP3 in individual cancer types.** All p-values of log-rank test are shown.


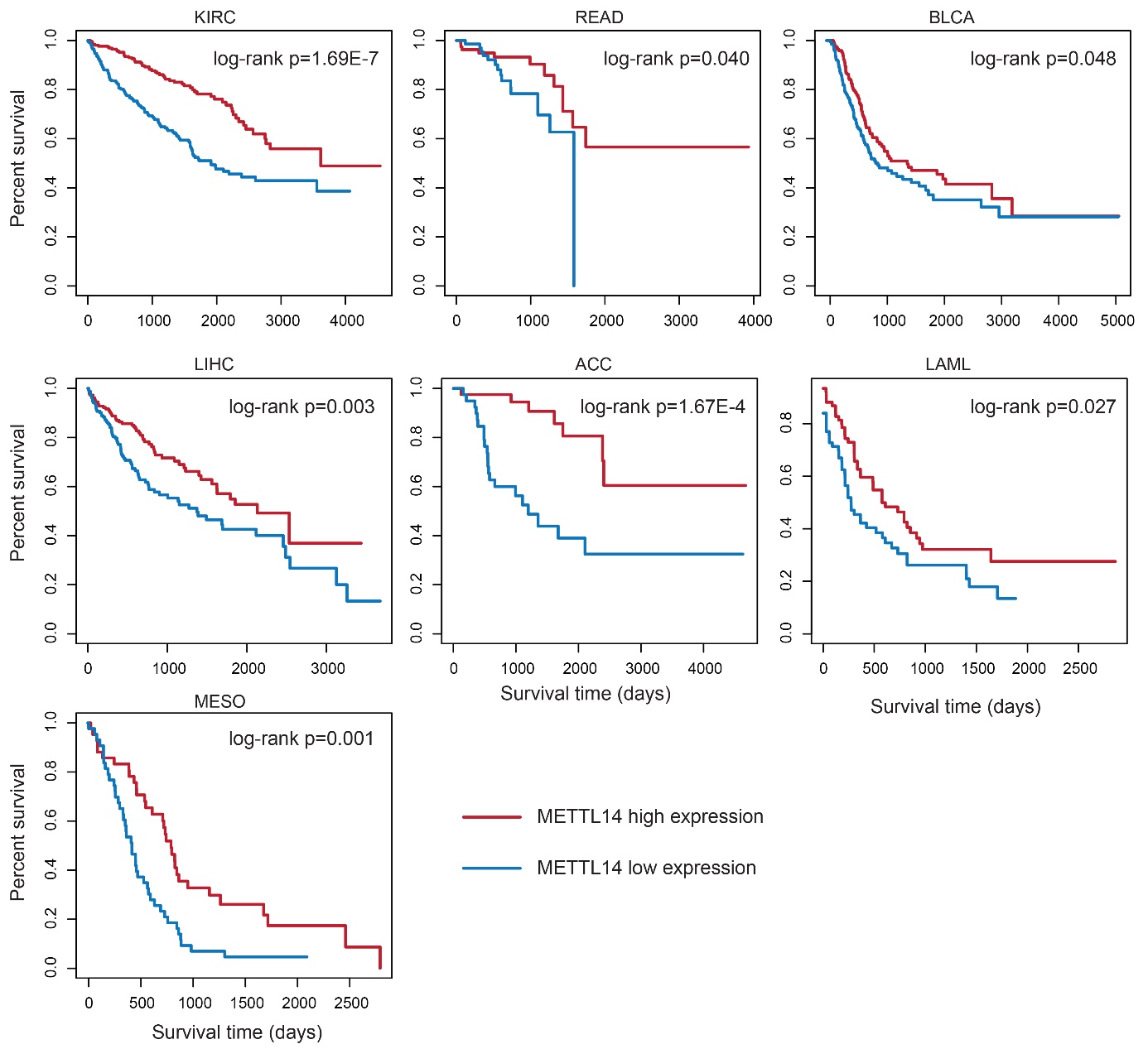


**Supplementary Fig. 8. Kaplan-Meier survival plots of patients grouped by the expression of METTL14 in individual cancer types.** All p-values of log-rank test are shown.


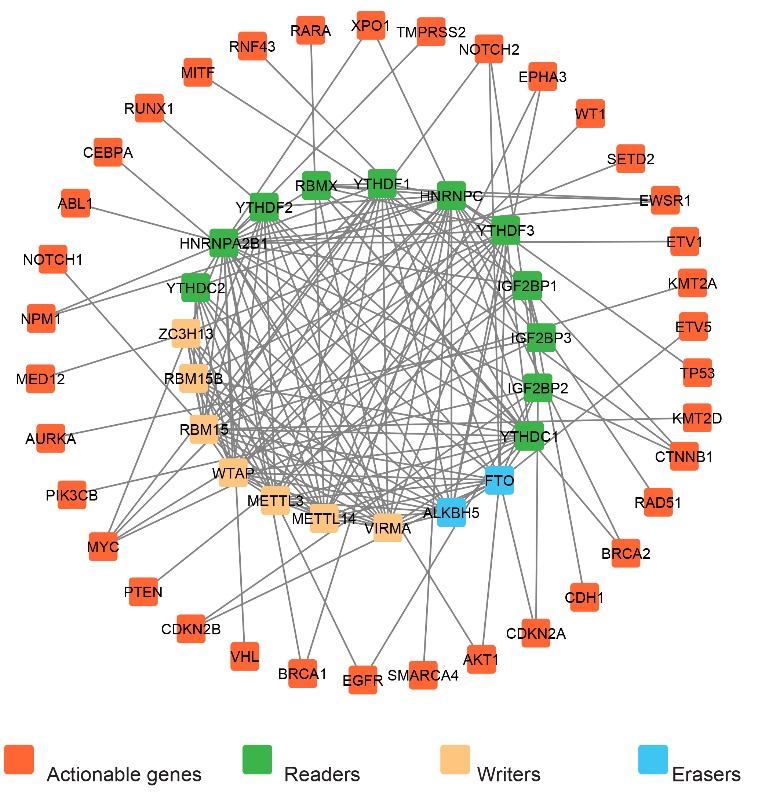


**Supplementary Fig. 9. Protein-protein interactions among m^6^A regulators and clinical actionable genes obtained from STRING database.** Genes are colored by different types.
